# The short-term effect of high versus moderate protein intake on recovery after strength training in resistance-trained individuals

**DOI:** 10.1186/s12970-017-0201-z

**Published:** 2017-11-21

**Authors:** Justin Roberts, Anastasia Zinchenko, Craig Suckling, Lee Smith, James Johnstone, Menno Henselmans

**Affiliations:** 1 Cambridge Centre for Sport and Exercise Sciences, Anglia Ruskin University, East Road, Cambridge, UK; 2 Kings College, University of Cambridge, Cambridge, UK; 3 Bayesian Bodybuilding R&D Department, Gorinchem, The Netherlands

**Keywords:** Protein timing, Strength performance, Training recovery

## Abstract

**Background:**

Dietary protein intakes up to 2.9 g.kg^−1^.d^−1^ and protein consumption before and after resistance training may enhance recovery, resulting in hypertrophy and strength gains. However, it remains unclear whether protein quantity or nutrient timing is central to positive adaptations. This study investigated the effect of total dietary protein content, whilst controlling for protein timing, on recovery in resistance trainees.

**Methods:**

Fourteen resistance-trained individuals underwent two 10-day isocaloric dietary regimes with a protein content of 1.8 g.kg^−1^.d^−1^ (PRO_MOD_) or 2.9 g.kg^−1^.d^−1^ (PRO_HIGH_) in a randomised, counterbalanced, crossover design. On days 8–10 (T1-T3), participants undertook resistance exercise under controlled conditions, performing 3 sets of squat, bench press and bent-over rows at 80% 1 repetition maximum until volitional exhaustion. Additionally, participants consumed a 0.4 g.kg^−1^ whey protein concentrate/isolate mix 30 min before and after exercise sessions to standardise protein timing specific to training. Recovery was assessed via daily repetition performance, muscle soreness, bioelectrical impedance phase angle, plasma creatine kinase (CK) and tumor necrosis factor-α (TNF-α).

**Results:**

No significant differences were reported between conditions for any of the performance repetition count variables (*p* > 0.05). However, within PRO_MOD_ only, squat performance total repetition count was significantly lower at T3 (19.7 ± 6.8) compared to T1 (23.0 ± 7.5; *p* = 0.006). Pre and post-exercise CK concentrations significantly increased across test days (*p* ≤ 0.003), although no differences were reported between conditions. No differences for TNF-α or muscle soreness were reported between dietary conditions. Phase angle was significantly greater at T3 for PRO_HIGH_ (8.26 ± 0.82°) compared with PRO_MOD_ (8.08 ± 0.80°; *p* = 0.012).

**Conclusions:**

When energy intake and peri-exercise protein intake was controlled for, a short term PRO_HIGH_ diet did not improve markers of muscle damage or soreness in comparison to a PRO_MOD_ approach following repeated days of intensive training. Whilst it is therefore likely that moderate protein intakes (1.8 g.kg^−1^.d^−1^) may be sufficient for resistance-trained individuals, it is noteworthy that both lower body exercise performance and bioelectrical phase angle were maintained with PRO_HIGH_. Longer term interventions are warranted to determine whether PRO_MOD_ intakes are sufficient during prolonged training periods or when extensive exercise (e.g. training twice daily) is undertaken.

## Background

Recent research suggests that resistance trainees benefit from increased training frequency [1] such as repeated bouts daily or across days. Increased training frequency stimulates muscle protein synthesis to a higher degree, which in turn, results in a greater anabolic potential. To sustain resistance training at high training frequencies, it is crucial for strength athletes to enhance their recovery ability. Several factors are advantageous for recovery and enhanced athletic performance in the following training sessions. For example, reduction in whole body protein breakdown following resistance training sessions has been demonstrated by the consumption of a meal high in protein [2]. Exercise-induced muscle damage that results in muscle soreness, reduces the ability to train and is potentially detrimental for the performance of a strength athlete [3]. Additionally, enhanced recovery is associated with a lower level of serum muscle damage and inflammation markers, such as creatine kinase and tumor necrosis factor alpha (TNF-α; [4–6]). The ability to recover can also be assessed by intense training sessions on consecutive days, which may lead to performance reduction over time [4, 7]. Previous research has suggested that peri-exercise protein ingestion is beneficial for muscle hypertrophy, strength gains and recovery after intense training sessions [8–10].

In one study, the efficacy of pre- and post-exercise whey protein ingestion on recovery from resistance exercise sessions in strength and power athletes was examined [4]. The researchers found that two servings of a protein blend containing 42 g protein each, consumed immediately before and after a strength training session, improved indices of recovery (CK levels, repetition performance) in resistance trained subjects compared to a maltrodextrin placebo. The protein intakes of the groups were 2.0 g.kg^−1^.d^−1^, excluding the supplements. Since energy intake and nutrient timing were not equated between groups, it is unclear if the faster recovery was the result of increased energy intake, the timing of the protein supplement or the protein intake itself. This critical question emerges from the findings of a multi-level meta-analysis which concluded that the total daily protein intake is the most important dietary variable for adaptations to resistance training and not protein timing around workouts [11]. This result may thus imply that very high levels of protein intake of about 2.9 g.kg^−1^.d^−1^, as ingested in the treatment group of the training recovery study [4], are required to optimize recovery to resistance training.

Numerous research studies have found no benefits for a total protein intake exceeding 1.8 g.kg^−1^.d^−1^ in resistance trained subjects [12–17], which is below the protein intake of 2.0 g.kg^−1^.d^−1^ consumed by the placebo group in the mentioned study [4]. For this reason, the aim of the current study was to investigate whether a high protein intake (2.9 g.kg^−1^) leading into and across repeated days of intensive training improves markers of recovery in resistance-trained individuals when both total energy intake and peri-exercise protein timing are controlled for. Our study will not only add to the research literature on whether protein intake can benefit acute training adaptations, but help to clarify the relative importance of protein intake on training recovery in relation to overall energy intake. We hypothesised that a short term high protein intake (2.9 g.kg^−1^) would not enhance recovery compared to a moderate protein intake (1.8 g.kg^−1^) in resistance trained individuals when energy intake and peri-exercise protein intake is controlled for.

## Methods

### Participants

This study was conducted in accordance with the Declaration of Helsinki, and the protocol was approved by the Faculty of Science and Technology Ethics Committee, Anglia Ruskin University (FST/FREP/15/556). Informed consent was obtained from all individual participants included in the study. Sample size was based on an a priori power analysis using G*Power (v.3.1.9.2, Dusseldorf [18]) to determine that 10 subjects were required to replicate the highest significant effect size of 1.05 for the between-group difference in performance [squat] repetitions (based on previous data [4]) using α = 0.05; 1-β = 0.80.

Participants were required to have a resistance training background of at least 18 months, and actively training >3 h per week at the time of inclusion. Additionally, all participants were required to achieve minimum lifting standards during baseline testing as follows: i) at least 55% of body weight (for women) and 110% of body weight (for men) for a standardised bench press test; ii) an ability to squat at least 100% of body weight (for women) and 150% of body weight (for men). All participants satisfactorily completed a health screen questionnaire, and had no known history of cardiovascular abnormalities, diabetes, or recent viral infections or injuries which would exclude them from repetitive training sessions.

Sixteen individuals (9 men, 7 women) volunteered for study inclusion, although two participants were excluded from the final analysis due to dietary non-compliance, resulting in 14 resistance trained participants in this randomised, controlled trial. None of the participants were using any anabolic substances, and were required to refrain from taking additional supplementation (e.g. creatine, beta-alanine) for 4 weeks prior to and during the study, to reduce conflict with the study parameters. Participant characteristics are displayed in Table 1.

**Table 1:** Participant characteristics and baseline measurements

Variable	All Participants (*n* = 14)	Male (*n* = 8)	Female (*n* = 6)
Age (years)	31 ± 6	30 ± 6	33 ± 6
Height (m)	1.71 ± 0.12	1.80 ± 0.45	1.60 ± 0.79 ***
Weight (kg)	78.45 ± 24.72	95.19 ± 18.98	56.13 ± 6.21 ***
FM (%)	17.47 ± 3.99	17.57 ± 4.81	17.33 ± 3.01
FM (kg)	14.13 ± 7.48	17.39 ± 8.49	9.78 ± 2.22
FFM (%)	82.53 ± 3.99	82.43 ± 4.81	82.67 ± 3.01
FFM (kg)	64.32 ± 18.42	77.79 ± 11.36	46.36 ± 4.86 ***
PhA (°)	8.19 ± 0.74	8.55 ± 0.37	7.72 ± 0.88 *
Squat 1RM (kg)	132.50 ± 53.67	167.19 ± 41.18	86.25 ± 24.99 ***
Bench Press 1RM (kg)	92.32 ± 43.16	124.38 ± 21.91	49.58 ± 18.33 ***
Row 1RM (kg)	85.71 ± 34.74	113.75 ± 11.26	48.33 ± 5.16 ***
CK (U^.^L^−1^)	172.92 ± 106.86	245.14 ± 92.59	88.67 ± 36.01 **
TNF-α (pg^.^mL^−1^)	1.54 ± 0.28	1.59 ± 0.26	1.47 ± 0.31

Outlines participant characteristics and baseline assessment measures. Reference to gender differences are also included to demonstrate adherence to inclusion criteria. Data are presented as M ± SD

*FM* Fat Mass, *FFM* Fat Free Mass, *PhA* Phase angle, *RM* repetition maximum, *CK* Creatine Kinase, *TNF-α* Tumor Necrosis Factor-α

Significant gender differences denoted as: *p = 0.03; **p = 0.003; ***p ≤ 0.001

### Procedures

#### Baseline measures

All testing took place within the Cambridge Centre for Sport and Exercise Sciences, Anglia Ruskin University, Cambridge. Participants were instructed to refrain from strenuous physical activity and consumption of caffeinated products ~48 h prior to baseline measures. Participants arrived acutely fasted (~3–4 h), after which body mass (Seca 780, Hamburg, Germany), height (Seca 200 stadiometer, Hamburg, Germany) and body composition were assessed under temperature controlled conditions. Body density was assessed via hydrostatic weighing (with water temperature maintained at 30 °C), from which total body fat, fat free mass and fat mass values were estimated using the Siri eq. (1961). For confirmation, total body fat was also evaluated using an 8 site skinfold calliper assessment (using guidelines outlined by the International Society for the Advancement of Kinanthropometry (ISAK)). All body composition measures were undertaken by the same researcher.

Following this, a venous wholeblood sample was collected from participants by a qualified phlebotomist into duplicate 4 ml K3EDTA vacutainers (Greiner Bio-One GmbH, Kremsmunster, Austria). Samples were centrifuged for 10 min at 3000 rpm, with aliquotted plasma pipetted into sterile, nonpyrogenic, polypropylene cyrovials (Fisherbrand, Fisher Scientific, Loughborough, UK) and immediately frozen at −80 °C for later assessment of creatine kinase (CK) and tumor necrosis factor alpha (TNF-α).

Following a 10 min rest period, participants undertook a 5 min warm up procedure on a Monark Ergomedic 874E cycle-ergometer before being assessed for maximal strength performance (1 repetition max – 1RM). All exercises were undertaken in the following order: squat, bench press, bent over row using a standard Olympic barbell (20 kg). For each exercise, participants undertook a warm up routine (10 repetitions at 40% predicted 1RM), before increasing to 5 repetitions at 60% predicted 1RM. A final set of 1–2 repetitions was undertaken at 80% 1RM, after which appropriate loading was undertaken to reach 1RM within 5 attempts. A rest period of ~5 min was permitted between 1RM attempts and main exercises. For the purposes of initial assessment, 15 min after the completion of the final 1RM assessment, a post exercise blood sample was collected in the same manner as described above. Baseline measures are displayed in Table 1, noting that inclusion criteria was met for both men (squat: 175.1 ± 19.5% and bench press: 131.8 ± 15.6% of body weight) and women (squat: 151.7 ± 30.5% and bench press: 87.0 ± 24.4% of body weight; M ± SD).

#### Dietary assessment

Prior to baseline measures, and throughout the intervention, participants were requested to maintain habitual food/activity diaries (following individual guidance in diary collation, with emphasis on meal content, portion size and weight, and fluid intake) using the smart phone app/ browser program MyFitnessPal. This method is a reliable food tracking method as described previously [17, 19, 20]. Diaries were assessed using Nutritics Professional Dietary Analysis software (Nutritics Ltd., Co. Dublin, Ireland). Initial assessment was used to monitor typical food choices and habitual caloric balance (including criterion assessment of training levels). From this, assessment of individual maintenance caloric intake was undertaken using the formula of Katch-McArdle (1996) based on an estimated resting daily energy expenditure (RDEE) of 370 + (21.6 * body weight in kg) and adjusted against training requirements and non-exercise adaptive thermogenesis [21], based on previous research [22]).

#### Experimental design and intervention

This study employed an experimental, randomised controlled, counter-balanced, crossover design. Participants were randomly assigned to a 10 day matched calorie period of either moderate (target: 1.8 g.kg^−1^.d^−1^) or high (target: 2.9 g.kg^−1^.d^−1^) total protein intake (PRO_MOD_ and PRO_HIGH_ respectively). At the end of the first dietary period, participants returned to habitual intake patterns for 24 h before beginning the opposing dietary condition. Throughout each intervention period, participants consumed a diet corresponding to maintenance requirements (using a macronutrient split (based on total calories) of ~40% carbohydrate, 25 or 35% protein and 25–35% fat) with a lead in period of 7 days prior to main testing. Guidance on meal intake, food options in line with habitual patterns and portion size was provided to each participant, along with provision of additional whey protein to supplement daily intake where required. Mean dietary intakes at baseline and for each intervention are shown in Table 2.

**Table 2:** Mean dietary intake at baseline and across intervention periods

Variable	Category	Baseline	PRO_MOD_	PRO_HIGH_
Energy Intake (EI)	(kcal^.^d^−1^)	2490.33 ± 496.04	2262.64 ± 495.78	2377.14 ± 509.97
	(kcal^.^kg^-1.^d^−1^)	31.82 ± 6.01	30.01 ± 4.87	31.43 ± 4.68
Protein Intake	(g^.^d^−1^)	174.00 ± 67.85*	140.36 ± 46.07	219.07 ± 69.90 ***
	(g^.^kg^-1.^d^−1^)	2.13 ± 0.57*	1.79 ± 0.11	2.81 ± 0.29 ***
	(%EI)	27.47 ± 7.77*	24.57 ± 4.81	36.46 ± 6.21 ***
Carbohydrate Intake	(g^.^d^−1^)	245.75 ± 74.89	243.86 ± 55.84	237.64 ± 68.65
	(g^.^kg^-1.^d^−1^)	3.16 ± 1.02	3.27 ± 0.68	3.19 ± 0.92
	(%EI)	39.15 ± 8.02	43.16 ± 2.81	39.94 ± 6.82
Fat Intake	(g^.^d^−1^)	83.25 ± 22.70**	80.64 ± 16.78	61.14 ± 12.86 ***
	(g^.^kg^-1.^d^−1^)	1.06 ± 0.26**	1.09 ± 0.23	0.82 ± 0.19 ***
	(%EI)	30.58 ± 7.04**	32.27 ± 2.79	23.60 ± 4.47 ***

Outlines average dietary intake at baseline and across intervention periods. Data are presented in absolute and relative categories. All data are presented as M ± SD

*PRO*_*MOD*_ moderate protein condition (target 1.8 g^.^kg^-1.^d^−1^), *PRO*_*HIGH*_ high protein condition (target 2.9 g^.^kg^-1.^d^−1^)

*Significantly different to PRO_HIGH_ only (*p* ≤ 0.002)

**Significantly different to PRO_HIGH_ only (*p* ≤ 0.014)

***Significant differences between intervention diet conditions (*p* ≤ 0.003)

During each 10 day period, participants maintained individual training routines, but were requested to refrain from strenuous training ~48 h prior to, and allow for sufficient rest the day before, main testing days. On days 8–10 of each dietary period, participants were required to attend the laboratory for main assessment sessions. For each assessment, participants arrived fasted from their previous meal (~3–4 h), prior to having body weight, bioelectrical impedance and resting blood measures undertaken as described. Single frequency bioelectrical impedance in a supine position (Impedimed DF50, Carlsbad, CA) was employed on testing days as a means to provide a proxy measure of muscle quality (membrane integrity) via phase angle (PhA) estimation.

Following this, participants were provided with a pre-exercise protein beverage comprising: 0.4 g.kg^−1^ whey protein concentrate/isolate mix (delivering 0.32 g.kg^−1^ protein; GoPro Whey Protein; High Quality whey protein complex, Go Protein Ltd., Langtoft, Peterborough, UK: containing (per 100 g) 408 kcal, 80 g protein (whey concentrate 85%, whey isolate 5%), 6.4 g fat and 7.5 g carbohydrate), 4 mg.kg^−1^ anhydrous caffeine (Myprotein Ltd., Cheshire, UK; to simulate typical pre-training practices) and 4 ml.kg^−1^ water; and allowed up to 5 min to consume. The whey protein mix contained (per 100 g) 8.3% leucine; 7.7% lysine; 5.2% threonine; 4.8% valine; 4.3% isoleucine; 2.3% phenylalanine; 1.7% methionine and 1.3% histidine.

On days 8–10 in each dietary period, participants were additionally assessed following beverage consumption for residual subjective muscle soreness. A 0–10 visual analogue scale (0 = none, 5 = noticeable, 7 = uncomfortable, 10 = severe) was employed for subjective assessment of soreness severity (registering both onset of ‘noticeable’ and ‘uncomfortable’ soreness) using a Wagner FDX digital algometer (Wagner Instruments, Greenwich, CT). Muscle soreness assessment was undertaken by the same researcher for consistency. Eight anatomical locations were assessed as a means to quantify global soreness estimates including: anterior deltoid, main pectoral, medial trapezius, main triceps, upper gluteus, upper and middle rectus femoris, vastus medialis with all measures taken on the right hand side of the body. Force applied was measured in newtons (N).

Thirty minutes after beverage consumption, all participants undertook strength exercise/assessment under supervision from qualified personnel. This comprised an initial warm up on the same cycle-ergometer as described followed by standard preparation sets similar to baseline (10 repetition at 40% 1RM, 5 repetitions at 60% 1RM) and 3 sets of 80% 1RM to failure. A period of 90 s was permitted between each set, and 5 min between main exercises (in the same order: squat, bench press, bent over row). The maximum number of completed repetitions with appropriate form was recorded. Verbal encouragement was provided by the same tester on all occasions in a standard manner. At the end of each testing period, participants rested for 15 min prior to a post assessment blood sample. At exactly 30 min post assessment, participants were provided with a second beverage containing 0.4 g.kg^−1^ whey protein complex (delivering 0.32 g.kg^−1^ actual protein) mixed with 4 ml.kg^−1^ water only.

#### Biochemical analyses

All samples were analysed by the Core Biochemical Analysis Laboratory (CBAL), Addenbrookes Hospital, Cambridge. Briefly, for CK, a bichromatic coupled enzyme reaction assay was employed using an automated Siemens Dimension® EXL analyser (Siemens Healthcare Diagnostics, Surrey, UK). The rate of nicotinamide adenine dinucleotide phosphate-oxidase (NADPH) production measured at 340 and 540 nm correlates with CK activity (analytical measurement range: 7–1000 U.L^−1^; intra-assay variance: 3.3% at 108 U.L^−1^, 1.7% at 788 U.L^−1^). TNF-α was assessed by a multiplexed electrochemical luminescence immunoassay (using the singleplex human proinflammatory panel 1 kit) on the MesoScale Discovery Sector S600 analyser (Meso Scale Diagnostics, Rockville, MD, US). Diluted recombinant human E.Coli was used to constitute a standard 8-point curve. A sulfo-tag anti-human TNF-α detection antibody was added to the sample, and the plate read following incubation and plate washing (quantitation range: 0.69–248 pg.mL^−1^; intra-assay variance: 3.4% at 4.45 pg.mL^−1^, 2.4% at 19.2 pg.mL^−1^, 2.7% at 75.5 pg.mL^−1^).

#### Statistical analyses

Statistical analyses were performed using SPSS (v20, IBM, Armonk, NY). Dependent variable distributions were assessed for normality with Shapiro-Wilk and Kolmogorov-Smirnov tests as well as manual inspections of M-estimators, histograms, stem-and-leaf plots and boxplots. Baseline gender differences and dietary intervention variables were assessed via a means comparison analysis of variance (ANOVA). A two-way repeated measures ANOVA with time (T1, T2, T3) and condition (PRO_MOD_, PRO_HIGH_) was performed for the main analyses, with Bonferonni post-hoc pairwise comparisons where applicable. An alpha level of ≤0.05 was employed for statistical significance. Data are reported as mean ± S.D.

## Results

### Dietary intakes

By standardising the PRO_MOD_ condition this resulted in an 18.4% reduction in daily protein intake compared to habitual levels, whereas the PRO_HIGH_ condition resulted in a 26.5% increase in typical protein intake for this cohort (see Table 2). Energy intake was comparable across both intervention periods (PRO_MOD_: 2262.64 ± 495.78 kcal.d^−1^ and PRO_HIGH_: 2377.14 ± 509.97 kcal.d^−1^; *p* > 0.05). Target protein amounts were closely met, with ratio intake being significantly higher with PRO_HIGH_ (2.81 ± 0.29 g.kg^−1^.d^−1^) compared with PRO_MOD_ (1.79 ± 0.11 g.kg^−1^.d^−1^; *p* = 0.0001). As carbohydrate intake did not significantly differ between groups for either total (g.d^−1^; *p* > 0.05) or ratio amounts g.kg^−1^.d^−1^ (*p* > 0.05), energy intake was balanced through a significantly lower fat intake with PRO_HIGH_ (61.14 ± 12.86 g.d^−1^) compared with PRO_MOD_ (80.64 ± 16.78 g.d^−1^; *p* = 0.002, see Table 2). Importantly, average body weight did not significantly differ between interventions (PRO_MOD_: 78.08 ± 24.24 kg v PRO_HIGH_: 78.28 ± 24.60 kg; *p* > 0.05) when trial order was considered.

### Repetition performance and recovery indices

For both the bench press and bent over row performances, no significant main effects or interactions were reported (*p* > 0.05; see Table 3). For squat repetitions, a significant time effect was observed (F = 3.905, *p* = 0.033, ƞp^2^ = 0.231), with post hoc analysis demonstrating that total repetitions were lower at T3 compared to T1 within PRO_MOD_ only (19.7 ± 6.8 v 23.0 ± 7.5 respectively, *p* = 0.006).

**Table 3:** Performance repetitions and global muscle soreness across testing days (T1-T3) for both moderate (PRO_MOD_) and high (PRO_HIGH_) protein dietary interventions

Variable	T1	T2	T3
*Squat*			
PRO_MOD_	23.0 ± 7.5	21.8 ± 8.4	19.7 ± 6.8***
PRO_HIGH_	22.3 ± 7.7	20.7 ± 8.3	20.1 ± 5.9
*Bench press*			
PRO_MOD_	17.0 ± 3.4	18.1 ± 3.8	18.3 ± 3.8
PRO_HIGH_	17.5 ± 2.9	17.4 ± 3.1	17.0 ± 3.6
*Bent over row*			
PRO_MOD_	20.2 ± 5.6	20.5 ± 6.7	21.4 ± 7.1
PRO_HIGH_	20.9 ± 7.4	20.6 ± 7.1	20.3 ± 7.2
*Muscle soreness (onset)*			
PRO_MOD_	69.3 ± 20.1	68.4 ± 16.9	70.2 ± 21.8
PRO_HIGH_	69.2 ± 22.4	65.3 ± 17.2	62.5 ± 17.9
*Muscle soreness (uncomfortable)*			
PRO_MOD_	88.1 ± 26.6	87.9 ± 26.8	88.2 ± 27.9
PRO_HIGH_	87.2 ± 26.8	82.9 ± 24.7	80.9 ± 26.0

Demonstrates performance repetitions and muscle soreness data relative to dietary interventions. Performance based on total repetitions across 3 sets. Global soreness based on average across 8 anatomical locations (N). All data are presented as M ± SD. ***significantly different from T1 for PRO_MOD_ only (*p* = 0.006)

**Fig. 1:**
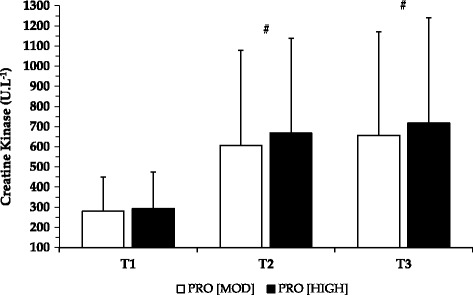
Mean creatine kinase (CK) levels post exercise for both dietary interventions. Figure demonstrates mean CK levels post exercise after each testing day (T1-T3). Data are presented as M ± SD. ^#^ Significantly different from T1 for both PRO_MOD_ and PRO_HIGH_ (*p* ≤ 0.003)

Resting pre-exercise creatine kinase (CK) concentrations at T1 were not significantly different compared to baseline levels (172.9 ± 106.86 U.L^−1^) for either PRO_MOD_ (227.40 ± 148.30 U.L^−1^) or PRO_HIGH_ (238.70 ± 164.00 U.L^−1^; *p* > 0.05). A significant time effect was, however, observed for resting CK (F = 20.313, *p* = 0.0001,ƞp^2^ = 0.610), with concentrations being higher at T3 (*p* = 0.001) and T2 (*p* = 0.002) compared to T1 for both conditions. No between group differences were observed. Post exercise CK concentrations are shown in Fig. 1. In a similar manner, whilst no main interactions were demonstrated, a significant main effect for time was noted (F = 18.194, *p* = 0.0001, ƞp^2^ = 0.583), with concentrations being higher at T3 (*p* = 0.003) and T2 (*p* = 0.002) compared to T1 for both conditions.

**Fig. 2:**
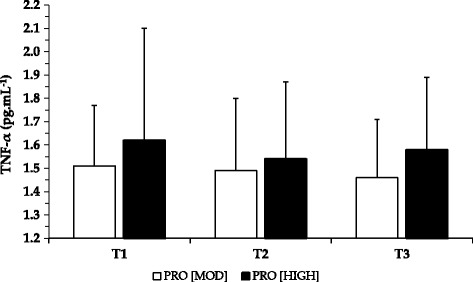
Mean tumor necrosis factor (TNF)-α levels post exercise for both dietary interventions. Figure demonstrates mean TNF-α levels following each testing day (T1-T3) for both dietary interventions. Data are presented as M ± SD. No significant differences were reported

Resting pre-exercise tumor necrosis factor-α (TNF-α) concentrations at T1 were comparable to baseline levels (1.52 ± 0.28 pg.mL^−1^) for both PRO_MOD_ (1.48 ± 0.29 pg.mL^−1^) and PRO_HIGH_ (1.65 ± 0.52 pg.mL^−1^; *p* > 0.05). Pre-exercise concentrations remained stable and within healthy ranges for both PRO_MOD_ (T2 = 1.49 ± 0.22 pg.mL^−1^; T3 = 1.54 ± 0.27 pg.mL^−1^) and PRO_HIGH_ (T2 = 1.61 ± 0.35 pg.mL^−1^; T3 = 1.62 ± 0.36 pg.mL^−1^). There was minimal influence of the strength training protocol, with no main or interaction effects reported on resting or post-exercise TNF-α concentrations across T1-T3. Post-exercise TNF-α concentrations are shown in Fig. 2.

**Fig. 3:**
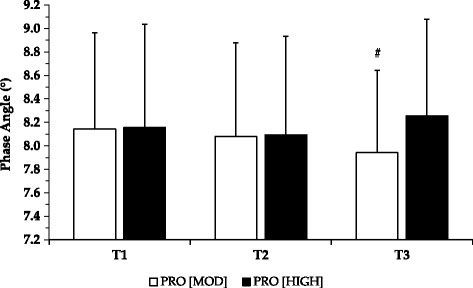
Pre-exercise phase angle assessment across testing days (T1-T3) for both dietary interventions. Figure shows the mean phase angle for both dietary interventions assessed pre-exercise across testing days. Phase angle assessed by bioelectrical impedance. All data are presented as M ± SD. ^#^ Significantly different between PRO_MOD_ and PRO_HIGH_ at T3 only (*p* = 0.012)

Mean onset of muscle soreness occurred earlier by 6.3% at T3 (62.5 ± 17.9 N) compared to T1 (69.2 ± 22.4 N), and by 5.1% for the ‘uncomfortable’ category across the same timeframe with PRO_HIGH_ (see Table 3). However, these observations were not significant within group. No main effects or interactions were found for muscle soreness categories. For phase angle (PhA) (see Fig. 3), whilst no main effects were found, a significant interaction between time and condition was reported (F = 4.044, *p* = 0.03, ƞp^2^ = 0.237). Post-hoc analysis showed that PhA was greater at T3 for PRO_HIGH_ (8.26 ± 0.82°) compared with PRO_MOD_ (8.08 ± 0.80°; *p* = 0.012).

## Discussion

The main aim of this study was to investigate whether a high protein intake (2.9 g.kg^−1^.d^−1^) leading into and across repeated days of intensive training improved markers of recovery in resistance-trained individuals when both total energy intake and peri-exercise protein timing were controlled for. Overall, recovery was comparable between dietary strategies when markers of muscle damage or soreness were considered. Similarly, performance repetitions were not found to be significantly different between dietary conditions, further indicating that PRO_MOD_ (1.8 g.kg^−1^.d^−1^) may be sufficient for resistance trained individuals. Our data are in line with a multitude of other studies finding no beneficial effects for strength trainees of consuming more than 1.8 g.kg^−1^.d^−1^ of protein [12–17, 23]. It is suggested, therefore, that the benefits observed in the previous study [4] were likely due to nutrient timing and not absolute protein intake.

Peri-exercise protein intake was an important parameter that was controlled for in this study. The concept of nutrient timing has previously been shown to have significant impact on muscular hypertrophy (cross-sectional area and lean mass gains) and maximal strength (upper and lower body) performance when a mixed protein drink was consumed pre-post exercise across a 10 week resistance training programme [9]. However, there is still some debate as to whether protein/energy intake post-exercise only is a more important contributing factor to net positive fractional synthetic rates (FSR; [24–27]) with or without carbohydrate intake. Previous research has indicated that consumption of whey protein (25 g) post exercise significantly augments myofibrillar FSR up to 5 h into recovery [28], suggesting that the consumption of protein during the acute recovery period is central to net protein synthesis.

Mechanistically, the inclusion of essential amino acids (EAAs) appear critical to potentiating a greater net protein synthesis over the 24 h recovery window [29]. In the current study, participants consumed 0.32 g.kg^−1^ of net protein before and after exercise (which amounted to an average intake of 25.1 ± 7.9 g net protein per serve) in line with dosages used in previous research [28]. Of the EAAs, L-leucine has been proposed to have significant influence on protein synthesis [30, 31] following resistance training (which in the current study was the dominant amino acid provided per serve). Current evidence infers that acute essential amino acid feeding may likely inactivate the tublerosclerosis complex, particularly tuberin (TSC2) leading to activation of mTOR and PDK1 pathways. This has bearing on key regulatory proteins during the initiation phase of myofibrillar resynthesis including: eukaryotic initiation factor 2 (eIF2), 4E binding proteins and the protein kinase S6 K1 [32]. Additionally, the concept of nutrient ‘sensing’ has been proposed in which other proteins (Vps34) may be key to stimulating mTOR/PDK1 synthesis pathways [32]. Minimising nutrient deprivation pre-exercise, and acute refeeding post exercise may therefore be required for maximal recovery gains (particularly when training frequency is considered). A possible reason why a recent meta-analysis [3] on this subject did not find any beneficial effect of nutrient timing is that the majority of included studies were performed on untrained individuals. The anabolic ‘window’ for untrained individuals may be prolonged for >2 days following resistance exercise in contrast to strength-trained individuals [1]. Additionally, in the majority of studies included in the meta-analyses, protein intakes (as well as protein timing) were not matched between the treatment and control groups.

It has been previously described that exercise intensity may alter protein requirements for athletes [33]. The exercise protocol applied in this study presented a realistic scenario of how strength athletes, especially powerlifters, train. Our study used a whole-body workout on three consecutive days in contrast to previous research [4] using a lower body protocol, in which an intense leg workout with 3 exercises was performed on the first day and then only the squat exercise on the following testing days. Additionally, we did not limit the repetition number to only 10 repetitions for each set, but encouraged the subjects to continue until volitional exhaustion which permitted a more intensive protocol over the testing days.

The increased difficulty level and muscle damaging potential of our exercise protocol was reflected in the CK values, which were ~4-times as high as previously reported [4] and exceeded the physiological range at T2 and T3. Elevated CK values 24 h or more after intense exercise have also been observed in previous research [4, 6, 34, 35]. Analogue to previous investigations [4], perceived muscle soreness was not significantly different between dietary conditions, despite earlier recorded onset of muscle soreness for PRO_HIGH_ at T2 and T3. This finding was unsurprising considering CK values were not significantly different between conditions, indicating that any myofibrillar damage due to the exercise protocol may have been comparable between dietary strategies.

Surprisingly, however, the exercise protocol did not influence TNF-α values. Previous studies implementing heavy lower body exercise protocols with resistance-trained individuals observed an increase in TNF-α immediately after exercise [5, 6]. In contrast, one research study measuring TNF-α response after an eccentric arm exercise protocol failed to observe significant changes in TNF-α [36]. The reason for this discrepancy may be that strenuous training of a smaller muscle group was not sufficient to elicit the same level of inflammatory response compared to larger muscle groups. Although our exercise protocol utilised a challenging whole body workout, it is also feasible that a significant elevation of TNF-α occurred >1–5 h after exercise, as reported elsewhere [37], or that the inclusion of a post-exercise protein formula may have blunted the TNF-α, but not the CK, response.

Overall, performance repetition scores across each testing day were not significantly different between dietary conditions. However, it is noteworthy that within condition lower body performance was maintained with PRO_HIGH_. In comparison, within condition only, squat performance significantly declined by T3 with PRO_MOD_ (despite no differences in overall number of repetitions performed throughout the assessment period between conditions: 64.5 ± 21.2 for PRO_MOD_ v 63.1 ± 19.4 for PRO_HIGH_; *p* = 0.477). Aligned with this, a significant interaction effect was found for bioelectrical impedance PhA, with values increasing at T3 for PRO_HIGH_ in contrast to PRO_MOD_. As PhA has been reported to be a proxy measure of muscle ‘quality’ [38–40], myofibrillar structure may have been maintained with PRO_HIGH_ which could have bearing for longer term performance gains during intensive periods of training. The results may indicate that a PRO_HIGH_ approach during repeated days of intensive exercise could support training maintenance pertinent to lower body exercise.

Previous research has shown that participation in a prolonged resistance training program is associated with an increase in PhA [40]. The mean PhA for athletes training for strength and power has also been reported to be higher than endurance athletes (8.4 ± 0.8 v 8.0 ± 1.0; [41]) indicating that PhA may depend on muscle fiber composition. To our knowledge, this is the first report of short-term changes in PhA as a result of repeated days of intensive resistance exercise coupled with modified protein intake. However, such findings should be interpreted with caution in light of the lack of significant differences between dietary groups for performance repetition scores and biomarkers of muscle damage. Additionally, such findings may only be applicable to strength-trained athletes, and may not necessarily apply to other sporting disciplines in which athletes train multiple times a day including sport specific and resistance training.

A further explanation for the lack of significant differences between dietary strategies for repetition performance may have been individual variability, which appeared to be particularly pronounced between men and women as reported elsewhere [42, 43]. Whereas some of the participants in this study were not able to perform more than 8 repetitions per set on the squat exercises, others were able to exceed this number by performing more than 15 repetitions at 80% of individual 1 RM. The muscle fiber composition of vastus lateralis is a genetic trait, which could explain 45% of the proportion of muscle composition, whereas ~40% can be explained by environmental factors e.g. a specific training protocol. For this reason, slow-twitch (Type I muscle fiber) content varies considerably (14–86%) between individuals [44]. Individuals with a higher slow-twitch muscle fiber content in the quadriceps have the genetic predisposition to perform more repetitions on the squat exercise, which likely influences the protocol intensiveness, overall post exercise muscle damage and potential net protein synthesis following both a PRO_MOD_ and/or PRO_HIGH_ diet.

Whilst the findings of this study indicate that a short term PRO_MOD_ approach may be sufficient to support markers of recovery in resistance-trained individuals undergoing repeated days of intensive exercise, the potential benefit of lower protein intakes (<1.8 g.kg^−1^.d^−1^) cannot be excluded. However, as it was noted that within group, lower body repetition performance significantly declined with PRO_MOD_ by the end of the assessment (along with reported differences in phase angle between dietary conditions), a lower protein intake may have resulted in further performance decrements. Future research on short-term lower protein intakes may be warranted to confirm this.

It is acknowledged that the acute nature of the dietary interventions and short-term cross over period may be study limitations. As participants in this study were experienced resistance-trained individuals who typically consumed protein intakes ~2.1 g.kg^−1^.d^−1^, a standardised approach to calorific intake in the week prior to the assessment period should have sufficed to evaluate whether total protein load influenced recovery across repeated training days. Whilst a longer wash-out period may have been beneficial, post-hoc assessment of potential order/carry-over effects revealed no overall significant differences for main variables or bodyweight between test periods. The dietary lead-in period prior to each assessment phase was therefore deemed satisfactory.

Participants were tested under the same conditions across assessment days, with peri-exercise protein intake and timing controlled for. Prior to each laboratory visit, participants were requested to maintain similar dietary patterns ensuring they were acutely fasted before arrival (3-4 h). However, individual variance in postprandial nutrient availability may have influenced study findings. Assessment in a longer term post-absorptive or overnight fasted state may have presented clearer findings. However, not only did our participants effectively act as their own controls by maintaining eating patterns prior to testing, but intensive training in an overnight fasted state may not have been realistic for such individuals.

Whilst the study design purposefully aimed to assess both male and female resistance-trained athletes, another limitation to the study was sample size (*n* = 14), which could result in the possibility of type II errors when interpreting the findings. Given that our sample size exceeded the a priori power analysis requirement of 10 subjects and that there was no significant effect between dietary conditions on any of the outcome measures (except phase angle), it is unlikely that the sample size masked a large effect of protein intake. Future research should consider evaluation of specific gender differences and overall training experience which may likely be confounding variables when assessing the impact of protein intake on recovery.

## Conclusions

A short term PRO_HIGH_ diet did not improve markers of muscle damage or soreness following repeated days of intensive training when daily calorie and peri-exercise protein intake was controlled for. The findings from this study indicate that moderate protein intakes (1.8 g.kg^−1^.d^−1^) may be sufficient for resistance-trained individuals during acute periods of intensive exercise. However, equivocally it is noteworthy that lower body exercise performance and bioelectrical phase angle were maintained with PRO_HIGH_. Longer term interventions are therefore warranted to determine whether PRO_MOD_ intakes are indeed sufficient during prolonged training periods or when extensive exercise (e.g. training twice daily) is undertaken with resistance-trained individuals.

## Abbreviations

ANOVA: Analysis of variance
CBAL: Core Biochemical Analysis Laboratory, Addenbrookes Hospital, Cambridge
CK: Creatine kinase
EAA: Essential amino acids
eIF2: eukaryotic initiation factor 2
mTOR: mechanistic target of rapamycin
N: Newtons
NADPH: Nicotinamide adenine dinucleotide phosphate-oxidase
PDK1: 3-phosphoinositide-dependent protein kinase
PRO_HIGH_: representative of the high protein (2.9 g.kg^−1^.d^−1^) condition
PRO_MOD_: representative of the moderate protein (1.8 g.kg^−1^.d^−1^) condition
RM: Repetition maximum
TNF-α: Tumor necrosis factor-α
TSC2: Tublerosclerosis complex 2, tuberin

## References

[1] Dankel SJ, Mattocks KT, Jessee MB, Buckner SL, Mouser J, Counts BR (2016). Frequency: the overlooked resistance training variable for inducing muscle hypertrophy?. Sports Med.

[2] Kim I-Y, Schutzler S, Schrader A, Spencer HJ, Azhar G, Ferrando A, Wolfe R (2015). The anabolic response to a meal containing different amounts of protein is not limited by the maximal stimulation of protein synthesis in healthy young adults. Am. J. Physiol.–Endocrin. Metab.

[3] Schoenfeld B, Contreras B (2013). Is postexercise muscle soreness a valid indicator of muscular adaptations?. Strength Cond J.

[4] Hoffman JR, Ratamess NA, Tranchina CP, Rashti SL, Kang J, Faigenbaum AD (2010). Effect of a proprietary protein supplement on recovery indices following resistance exercise in strength/power athletes. Amino Acids.

[5] Townsend JR, Fragala MS, Jajtner AR, Gonzalez AM, Wells AJ, Mangine, GT et al. β-Hydroxy-β-methylbutyrate (HMB)-free acid attenuates circulating TNF-α and TNFR1 expression post-resistance exercise. J Appl Physiol 2013; 115 (8): 1173–1182. doi:10.1152/japplphysiol. 00738.2013. 10.1152/japplphysiol.00738.2013 23908318

[6] Townsend JR, Hoffman JR, Fragala MS, Jajtner AR, Gonzalez AM, Wells AJ (2015). TNF-α and TNFR1 responses to recovery therapies following acute resistance exercise. Front Physiol.

[7] Rowlands DS, Rössler K, Thorp RM, Graham DF, Timmons BW, Stannard SR, Tarnopolsky M (2008). Effect of dietary protein content during recovery from high-intensity cycling on subsequent performance and markers of stress, inflammation, and muscle damage in well-trained men. App Physiol Nutr Metab.

[8] Esmarck B, Andersen JL, Olsen S, Richter EA, Mizuno M, Kjaer M (2001). Timing of post exercise protein intake is important for muscle hypertrophy with resistance training in elderly humans. J Physiol.

[9] Cribb P, Hayes A (2006). Effects of supplement-timing and resistance exercise on skeletal muscle hypertrophy. Med Sci Sports Exerc.

[10] Hulmi JJ, Laakso M, Mero AA, Häkkinen K, Ahtiainen JP, Peltonen H (2015). The effects of whey protein with or without carbohydrates on resistance training adaptations. J Int Soc Sports Nutr.

[11] Schoenfeld BJ, Aragon AA, Krieger JW (2013). The effect of protein timing on muscle strength and hypertrophy : a meta-analysis. J. Int. Soc. Sports Nutr..

[12] Tarnopolsky MA, MacDougall JD, Atkinson SA (1988). Influence of protein intake and training status on nitrogen balance and lean body mass. J. App. Physiol..

[13] Tarnopolsky MA, Atkinson SA, MacDougall JD, Chesley A, Phillips S, Schwarcz HP (1992). Evaluation of protein requirements for trained strength athletes. J. App. Physiol.

[14] Lemon PW, Tarnopolsky MA, MacDougall JD, Atkinson SA (1992). Protein requirements and muscle mass/strength changes during intensive training in novice bodybuilders. J. App. Physiol..

[15] Hoffman JR, Ratamess NA, Kang J, Falvo MJ, Faigenbaum AD (2006). Effect of protein intake on strength, body composition and endocrine changes in strength/power athletes. J. Int. Soc. Sports Nutr..

[16] Hoffman JR, Ratamess NA, Tranchina CP, Rashti SL, Kang J, Faigenbaum AD (2009). Effect of protein-supplement timing on strength, power, and body-composition changes in resistance-trained men. Int J Sport Nutr Exerc Metab.

[17] Antonio J, Peacock C, Ellerbroek A, Fromhoff B, Silver T (2014). The effects of consuming a high protein diet (4.4 g/kg/d) on body composition in resistance-trained individuals. J. Int. Soc. Sports Nutr..

[18] Faul F, Erdfelder E, Lang A-G, Buchner A (2007). G*power 3: a flexible statistical power analysis program for the social, behavioral, and biomedical sciences. Behav Res Meth.

[19] Antonio J, Ellerbroek A, Silver T, Orris S, Scheiner M, Gonzalez A, diet PCA h p (2015). (3.4 g/kg/d) combined with a heavy resistance training program improves body composition in healthy trained men and women – a follow-up investigation. J. Int. Soc. Sports Nutr..

[20] Antonio J, Ellerbroek A, Silver T, Vargas L, Peacock C (2016). The effects of a high protein diet on indices of health and body composition – a crossover trial in resistance-trained men. J. Int. Soc. Sports Nutr..

[21] McArdle W, Katch F, Katch V (2001). Exercise physiology.

[22] Cunningham J (1982). Body composition and resting metabolic rate: the myth of feminine metabolism. Am J Clin Nutr.

[23] Walberg JL, Leidy MK, Sturgill DJ, Hinkle DE, Ritchey SJ, Sebolt DR (1988). Macronutrient content of a hypoenergy diet affects nitrogen retention and muscle function in weight lifters. Int J Sports Med.

[24] Mori H (2014). Effect of timing of protein and carbohydrate intake after resistance exercise on nitrogen balance in trained and untrained young men. J Physiol Anthro.

[25] Smiles WJ, Hawley JA, Camera DM (2016). Effects of skeletal muscle energy availability on protein turnover responses to exercise. J Exp Biol.

[26] Murphy CH, Churchward-Venne TA, Mitchell CJ, Kolar NM, Kassis A, Karagounis LG, Burke LM, Hawley JA, Phillips SM (2015). Hypoenergetic diet-induced reductions in myofibrillar protein synthesis are restored with resistance training and balanced daily protein ingestion in older men. Am J Physiol Endocrinol Metab.

[27] Burk A, Timpmann S, Medijainen L, Vahi M, Oopik V (2009). Time-divided ingestion pattern of casein-based protein supplement stimulates an increase in fat-free body mass during resistance training in young untrained men. Nutr Res.

[28] Moore DR, Tang JE, Burd NA, Rerecich T, Tarnopolsky MA, Phillips SM (2009). Differential stimulation of myofibrillar and sarcoplasmic protein synthesis with protein ingestion at rest and after resistance exercise. J Physiol.

[29] Tipton KD, Ferrando AA, Phillips SM, Doyle D, Wolfe RR (1999). Postexercise net protein synthesis in human muscle from orally administered amino acids. Am J Phys.

[30] Dreyer HC, Drummond MJ, Pennings B, Fujita S, Glynn EL, Chinkes DL (2008). Leucine-enriched essential amino acid and carbohydrate ingestion following resistance exercise enhances mTOR signalling and protein synthesis in human muscle. Am J Physiol Endocrinol Metab.

[31] Drummond MJ, Rasmussen BB (2008). Leucine-enriched nutrients and the regulation of mammalian target of rapamycin signalling and human skeletal muscle protein synthesis. Curr Opin Clin Nutr Metab Care.

[32] Barr K (2006). Training for endurance and strength: lessons from cell signaling. Med Sci Sports Exerc.

[33] Fielding RA, Parkington J (2002). What are the dietary protein requirements of physically active individuals? New evidence on the effects of exercise on protein utilization during post-exercise recovery. Nutrition in Clinical Care: An Official Publication of Tufts University.

[34] Kraemer WJ, Hatfield DL, Spiering BA, Vingren JL, Fragala MS, Ho J-Y (2007). Effects of a multi-nutrient supplement on exercise performance and hormonal responses to resistance exercise. Eur J Appl Physiol.

[35] Rawson ES, Conti MP, Miles MP (2007). Creatine supplementation does not reduce muscle damage or enhance recovery from resistance exercise. J Strength Cond Res.

[36] Serravite DH, Perry A, Jacobs KA, Adams JA, Harriell K, Signorile JF (2014). Effect of whole-body periodic acceleration on exercise-induced muscle damage after eccentric exercise. Int J Sports Physiol Perform.

[37] Levitt DE, Duplanty AA, Budnar RG, Luk H-Y, Fernandez A, Layman TJ (2015). The effect of post-resistance exercise alcohol ingestion on lipopolysaccharide-stimulated cytokines. Eur J Appl Physiol.

[38] Barbosa-Silva MCG, Barros AJD, Wang J, Heymsfield SB, Pierson RN (2005). Bioelectrical impedance analysis: population reference values for phase angle by age and sex. Am J Clin Nutr.

[39] Norman K, Stobäus N, Pirlich M, Bosy-Westphal A (2012). Bioelectrical phase angle and impedance vector analysis - clinical relevance and applicability of impedance parameters. Clin Nutr.

[40] Souza MF, Tomeleri CM, Ribeiro AS, Schoenfeld BJ, Silva AM, Sardinha LB, Cyrino ES (2016). Effect of resistance training on phase angle in older women: a randomized controlled trial. Scand J Med Sci Sports.

[41] Koury JC, Torres AG, Trugo NMF (2014). Phase angle and body impedance vectors in adolescent and adult male athletes. Int. J. Sports Physiol. Perform..

[42] Maughan RJ, Harmon M, Leiper JB, Sale D, Delman A (1986). Endurance capacity of untrained males and females in isometric and dynamic muscular contractions. Eur J App Physiol Occ Physiol.

[43] Hunter SK (2014). Sex differences in human fatigability: mechanisms and insight to physiological responses. Acta Physiol.

[44] Simoneau J-A, Bouchard C (1995). Genetic determinism of fiber type proportion human skeletal muscle. FASEB J.

